# Space Radiation Biology for “Living in Space”

**DOI:** 10.1155/2020/4703286

**Published:** 2020-04-08

**Authors:** Satoshi Furukawa, Aiko Nagamatsu, Mitsuru Nenoi, Akira Fujimori, Shizuko Kakinuma, Takanori Katsube, Bing Wang, Chizuru Tsuruoka, Toshiyuki Shirai, Asako J. Nakamura, Asako Sakaue-Sawano, Atsushi Miyawaki, Hiroshi Harada, Minoru Kobayashi, Junya Kobayashi, Takekazu Kunieda, Tomoo Funayama, Michiyo Suzuki, Tatsuo Miyamoto, Jun Hidema, Yukari Yoshida, Akihisa Takahashi

**Affiliations:** ^1^Japan Aerospace Exploration Agency, 2-1-1 Sengen, Tsukuba, Ibaraki 305-8505, Japan; ^2^National Institute of Radiological Sciences, National Institutes for Quantum and Radiological Science and Technology (QST), 4-9-1 Anagawa, Inage-ku, Chiba 263-8555, Japan; ^3^Department of Biological Sciences, College of Science, Ibaraki University, 2-1-1, Bunkyo, Mito, Ibaraki 310-8512, Japan; ^4^Lab for Cell Function and Dynamics, CBS, RIKEN, 2-1 Hirosawa, Wako, Saitama 351-0198, Japan; ^5^Radiation Biology Center, Graduate School of Biostudies, Kyoto University, Yoshida Konoe-cho, Sakyo-ku, Kyoto 606-8501, Japan; ^6^Department of Biological Sciences, Graduate School of Science, The University of Tokyo, 7-3-1 Hongo, Bunkyo-ku, Tokyo 113-0033, Japan; ^7^Takasaki Advanced Radiation Research Institute, QST, 1233 Watanuki-machi, Takasaki, Gunma 370-1292, Japan; ^8^Research Institute for Radiation Biology and Medicine, Hiroshima University, Kasumi 1-2-3, Minami-ku, Hiroshima 734-8553, Japan; ^9^Graduate School of Life Sciences, Tohoku University, 2-1-1 Katahira, Aoba-ku, Sendai, Miyagi 980-8577, Japan; ^10^Division for the Establishment of Frontier Sciences of the Organization for Advanced Studies, Tohoku University, 2-1-1 Katahira, Aoba-ku, Sendai, Miyagi 980-8577, Japan; ^11^Gunma University Heavy Ion Medical Center, 3-39-22 Showa-machi, Maebashi, Gunma 371-8511, Japan

## Abstract

Space travel has advanced significantly over the last six decades with astronauts spending up to 6 months at the International Space Station. Nonetheless, the living environment while in outer space is extremely challenging to astronauts. In particular, exposure to space radiation represents a serious potential long-term threat to the health of astronauts because the amount of radiation exposure accumulates during their time in space. Therefore, health risks associated with exposure to space radiation are an important topic in space travel, and characterizing space radiation in detail is essential for improving the safety of space missions. In the first part of this review, we provide an overview of the space radiation environment and briefly present current and future endeavors that monitor different space radiation environments. We then present research evaluating adverse biological effects caused by exposure to various space radiation environments and how these can be reduced. We especially consider the deleterious effects on cellular DNA and how cells activate DNA repair mechanisms. The latest technologies being developed, e.g., a fluorescent ubiquitination-based cell cycle indicator, to measure real-time cell cycle progression and DNA damage caused by exposure to ultraviolet radiation are presented. Progress in examining the combined effects of microgravity and radiation to animals and plants are summarized, and our current understanding of the relationship between psychological stress and radiation is presented. Finally, we provide details about protective agents and the study of organisms that are highly resistant to radiation and how their biological mechanisms may aid developing novel technologies that alleviate biological damage caused by radiation. Future research that furthers our understanding of the effects of space radiation on human health will facilitate risk-mitigating strategies to enable long-term space and planetary exploration.

## 1. Introduction

Yuri Gagarin was the first human to journey into outer space. He completed one orbit of Earth on 12 April 1961. Almost 60 years have passed since this event, and space mission durations have remarkably extended. Currently, it is possible for humans to spend more than 6 months in outer space on the International Space Station (ISS). The ISS circles the Earth at an altitude of approximately 400 km. The living environment on the ISS is challenging to astronauts because microgravity (*μG*) induces musculoskeletal atrophy, isolated and limited habitability causes psychological stress, and exposure to space radiation potentially endangers the health of the astronauts [1].

The next challenging steps for humankind include new missions to the Moon followed by human exploration of Mars. In a Mars mission, the long distance between Earth and Mars will make the total mission duration 800–1,100 days, of which approximately 500 days will be spent on the surface of the planet, depending on the final mission design [2]. As a result, radiation exposure is expected to be greater when compared with that of a 6-month mission on the ISS. One major health concern in such prolonged missions is the amount of radiation exposure that accumulates over the duration of the lives of the astronauts. Therefore, health risks associated with exposure to space radiation are an important topic in a human Mars mission. The focus of this review is space radiation. We will initially discuss the environment of space radiation. This will be followed by a description of the various kinds of research endeavors undertaken to evaluate and minimize adverse biological outcomes caused by space radiation exposure.

## 2. Environment of Space Radiation

### 2.1. Radiation Environment in Low-Earth Orbits (LEO)

As mentioned in previous reviews [3–6], important ionizing radiation (IR) sources in the ISS orbits (altitude: 300 to 400 km; orbital inclination: 51.6°) include the three primary radiation sources (galactic cosmic rays (GCRs), which range widely from protons to Fe-ions, solar particle events (SPEs), and electrons and protons trapped in the Van Allen Belts (TPs)) outside the spacecraft. These combine to produce a complex radiation environment in and around the ISS, and the complexity of this radiation is dependent on the solar cycle, altitude, and shielding of each module of the ISS.

Primary GCRs comprise protons and high-energy heavy-ion (HZE) charged particles with energy spectra forming a broad peak around 1 GeV/n [3]. Fluxes of less than about 10 GeV/n are inversely related to solar activity [7]. The GCR fluxes depend heavily on the ISS altitude and are difficult to shield against using a realistic shielding mass for the ISS structure because of their high energy. Primary GCRs produce many secondary particles through projectile and target fragmentation in the ISS shielding materials and in the bodies of astronauts. The fluxes of primary TPs increase substantially as the altitude of the ISS increases [3, 7–9]. Although the fluxes of primary TPs can be effectively reduced by thin shielding (a few g/cm^2^), secondary particles produced by nuclear reactions increase in number as shielding mass increases and become dominant in fluxes under thick shielding conditions [10, 11]. Thus, TPs play a role in increasing or decreasing the exposure of astronauts to radiation in LEO. The energies of TPs are generally lower than those of GCRs, and their maximum energy is approximately several hundred MeV.

Since the construction of ISS began in 1998, there have been more than 120 SPEs (counted by NOAA, Space Weather Prediction) that have affected the Earth environment over solar cycles 23 to 24. The emergency return of astronauts following flight rules [12] due to severe SPEs has never occurred before because of Earth's protective magnetic field.

Japan Aerospace Exploration Agency (JAXA) has conducted a series of monitoring experiments to evaluate the radiation environment inside and outside the Japanese Experiment Module Kibo, which is part of the ISS with Passive Dosimeter for Life-Science and Experiments in Space (PADLES) [9, 13, 14]: area radiation monitoring in the Japanese Experiment Module “Kibo” of the ISS (Area PADLES) [15]; dose measurements of biological samples exposed to space radiation (Bio PADLES) [16–19]; radiation dosimetry of Asian astronauts (Crew PADLES); various kinds of international cooperative experiments with ISS partners (DosimetricPADLES); measurement of the directional dependence of the radiation dose inside the Kibo module (Exp PADLES) [11, 20]; and measurement of outside doses and evaluation of the shielding effect of the ISS Kibo hull (Free-space PADLES). Those experiments were initiated in 2008 just after attachment of the Japanese Pressurized Module (JPM) to the ISS.

We concluded that the characteristics of the space radiation environment in LEO contain the following: (i) a high contribution from high-linear energy transfer (LET) radiation that have a high-quality factor (QF) up to 30; (ii) dose rates have values that are a few hundred times greater than those on the ground; (iii) the directional distribution of space radiation is nearly isotropic; and (iv) radiation effects occur under *μG* [13, 14, 19]. Space radiation for LET greater than several keV/*μ*m causes more serious damage to living things than low-LET radiation. Measurements only of absorbed doses are insufficient for investigating biological effects or assessing radiation risk to astronauts. Dose equivalents taking into account the LET distributions of high-LET particles, their high radiation QFs, and relative biological effectiveness (RBE) must be measured considering space radiation environment.

Space radiation environments include fast neutrons with a wide energy range beyond several tens of MeV. The neutron dose contribution has been roughly estimated through the STS-89 space shuttle mission/Mir experiment with RRMD-III for charged particles and BBND for neutrons with energies less than 15 MeV, both loaded simultaneously [21]. Neutron doses contributing to total doses in LEO and around the Moon and Mars are still being estimated with various simulation codes. However, no practical measurement has been established so far with a neutron personal dosimeter applicable to energy exceeding ~20 MeV. The most physical and practical approach for estimating the high-energy neutron dose is to theoretically and experimentally determine LET values of energetic charged particles released by interactions with the neutrons and an anthropomorphic phantom. The dose-equivalent part of the practical dose can be obtained using the relation between QFs and LET values *via* the Q–L relation ICRP 60 [22]. Therefore, dose equivalents taking into account the LET distributions are also important for evaluating neutron doses.

### 2.2. Radiation Environment beyond LEO (Deep Space, the Moon, and Mars)

The space radiation environment differs in and beyond LEO, including the surface of the Moon [23–28], Mars [23], deep space [29, 30], and their comparisons [23, 31]. In past explorations, space radiation measurements have been conducted by three interplanetary missions in the orbital environment of both the Moon and Mars to generate global dosage maps and to measure energy spectra below 100 MeV [32–36]. In deep space outside Earth's protective magnetic field, HZE charged particles of GCRs and solar energetic particles (SEPs) strongly affect the dosimetry of astronauts. Space radiation doses change drastically because of the varying intensity and peak amplitude of SEP events in and near the Moon and Mars environments, where a protective magnetic field is almost completely absent.

Therefore, for radiation dose management of astronauts exposed to both SEPs and GCRs, it is essential to establish methods for estimating organ doses and effective doses that are both relative to career dose limits. These are obtained from the energy spectra of space radiation and doses from personnel dosimeters and environmental radiation monitoring systems.

Currently, as part of the NASA Artemis program, astronauts will land on the Moon by 2024. Under the umbrella of Artemis, the Lunar Orbital Platform-Gateway, which is a station orbiting the Moon, provides an international cooperation platform for scientific experiments and exploration of the lunar surface. The career dose limits for gateway are still under coordination between international partners. Currently, there is no interplanetary mission to measure the space environment in Japan. Thus, we must conduct actual measurements beyond LEO to determine effective materials, effective locations, and appropriate thicknesses or combinations on the basis of benchmark evaluations. This information will be useful for interplanetary space flight and travel expected in the near future.

### 2.3. Solar Ultraviolet (UV) Radiation

UV is part of the natural energy produced by the sun. UV radiation has electromagnetic radiation wavelengths from 10 nm to 400 nm, which are shorter than visible light (400–700 nm) but longer than X-ray. UV radiation reaches the Earth surface. UV radiation is classified into three regions based on their effects on biological processes: UV-C (<280 nm), UV-B (280–315 nm), and UV-A (315–400 nm). UV-C, which is a highly energetic wavelength, is eliminated by the stratospheric ozone layer and is not encountered by plants. Both UV-B and UV-A radiations reach the surface of Earth [37].

Cyanobacteria have created a foundation of the environment in which most organisms live today. The ozone layer completely absorbs harmful UV-C radiation (<285 nm), and through evolution, organisms were able to expand their habitat from water to land. The first land organisms, which resembled the liverworts, have evolved into the diverse range of plant species that exist. Sunlight-driven photosynthesis maintained the composition of the atmosphere, and plants serve as a food source for animals. Although sunlight is highly beneficial for life on Earth, it contains harmful UV-B radiation (280–315 nm) despite its efficient absorption by the ozone layer [38]. Although UV-B radiation accounts for <0.5% of the total solar energy on the surface of the Earth, its high energy causes damage to important cellular components, such as DNA, RNA, protein, and lipids, as it is readily absorbed by such macromolecules [39]. Among them, DNA, which stores genetic information, is a major target of UV-induced damage, and UV radiation can directly alter its structure. The main UV-induced photoproducts are cyclobutane pyrimidine dimers (CPDs) and pyrimidine-pyrimidone (6-4) photoproducts, which are also termed (6-4) photoproducts, and form between adjacent pyrimidines on the same strand [39, 40]. CPDs account for approximately 75% of DNA damage and the (6-4) photoproducts for the majority of the remaining 25%. DNA damage impedes replication and transcription, induces mutations, and may be lethal [39, 40]. Therefore, UV radiation causes damage to all organisms including plants. Most skin cancers are caused by UV radiation damaging the DNA in skin cells. Plants, which are sessile organisms, are at a higher risk of UV-B damage in comparison with motile organisms, which reduces growth and productivity.

The environment of space is characterized by low gravity, temperature oscillation, short-wavelength solar UV radiation, and complex cosmic IR. In particular, space is showered by a variety of different types of radiation, and thus, astronauts are exposed to a considerably large amount of space radiation [41, 42]. Moreover, in space, UV-C with shorter wavelengths than UV-B are much more prevalent, and its intensity is much higher than on Earth. On the surface of Mars, the UV-B radiation is remarkably higher than that on Earth and exceeds the safety limit for terrestrial life [43, 44]. Therefore, to establish sustainable life support systems for securing long-term human life in space, the effects of the complicated space environment not only for humans but also for plants must be understood. The growth and survival of plants will be required to supply nutrients and oxygen to humans under a resource-recycling system in space.

## 3. Irradiation Tests with Ground Facilities Similar to the Environment in Space

### 3.1. Low Dose Rate Irradiation Facilities

Humans are continuously exposed to low doses of background radiation and may also be exposed to low doses of IR from X-ray or CT scans and occupational usage of radiation as medical doctors, radiologists, or nuclear power plant workers. Residents in high background radiation areas or space station astronauts are exposed to low dose rates of IR for long periods. Residents in the vicinity of the evacuated areas of Chernobyl and Fukushima Daiichi nuclear power plant disasters may also have been exposed to low dose rates of IR and have health risk concerns because of exposure to above-average levels of IR. Biological responses toward acute irradiation from high doses of IR have been well characterized, and the molecular mechanisms of cell cycle checkpoints and DNA repair have been studied extensively. However, the biological effects and the health risks with low dose or low-dose-rate radiation exposure remain poorly understood. Understanding the health risks (mainly cancer) due to low doses of IR has been provided by epidemiological studies of atomic bomb survivors [45]; however, the risk or biological effects from chronic exposure to low dose rates of IR has only recently been more examined. To understand the molecular mechanisms following exposure to low dose rates of IR, irradiation instruments for chronic exposure to low dose rates of IR have been established in Japan and other countries [46]. The instruments in Japan use ^137^Cs as a radiation source and can irradiate biological specimens with *γ*-rays. Among them, the irradiation instrument for the Institute for Environmental Sciences (IES) can expose small animals such as mice with extremely low dose rates (about 0.05 mGy/day), and other instruments at IES can expose mice to different low dose rates (about 1 or 20 mGy/day). These instruments can perform chronic irradiation for a few years and have been supplying important information on the biological effects of chronic low dose rate irradiation to mice [46]. The instruments at the Research Institute for Radiation Biology and Medicine (RIRBM), Hiroshima University, Central Research Institute of Electric Power Industry (CRIEPI), University of Occupational and Environmental Health (UOEH), and National Institute of Radiological Sciences, National Institutes for Quantum and Radiological Science and Technology (QST-NIRS), can also perform chronic irradiation with dose rates that are higher than the instruments at IES. The instruments at CRIEPI and UOEH can irradiate cultured cells, and IES and RIRBM possess irradiation instruments for exclusive use on cultured cells. The chronic irradiation instrument at the Radiation Biology Center (RBC), Graduate School of Biostudies, Kyoto University, can be used to irradiate cultured cells and small fish [47]. The instruments have three different ^137^Cs radiation sources and stands (where the CO_2_ incubator for culture cells or aquarium for small fish is placed on) that can be placed at distances between 1.3 and 12 m from the radiation source. The machine exposes cultured cells or small fish to *γ*-rays over the range of 0.3–1,500 mGy/day by combining the radiation source choice and distance. The dose rate of space radiation in the ISS is 0.5 mGy/day [13–19]. Thus, the instrument at the RBC may provide important information about the health risks to astronauts at the ISS. The use of such chronic irradiation instruments in Japan is expected to provide important information to clarify the biological effects and health risks to humans under various chronic low dose rates of irradiation.

### 3.2. High-Energy Particle Irradiation Facilities

Dose rates from cosmic radiation such as SEP and GCR are low at around 0.5 mGy/day as measured inside ISS Kibo [13–19]. Sometimes this increases to tens of mGy/day during SPE events lasting up to several days. However, this will become a serious health issue over long stay periods in future missions to Mars and other planets. SEP and GCR contain various radiations, including *γ*-ray, electron, neutron, proton, and heavier ions. In particular, GCR includes heavier ions up to Fe (*Z* = 26), and these heavier ions can significantly affect crew and electric devices in a manned spacecraft because of their high ionization density. The interesting energy range of GCR is from 100 MeV/u to several GeV/u. This energy spectrum is around the peak flux of GCR and difficult to shield against in a spacecraft. Experiments involving cosmic radiation in space are expensive and rather time-limited. Although it is difficult to reproduce the cosmic radiation environment on the ground, experiments using HZE accelerators are important.

The pioneer of radiation research of HZEs was Bevalac at the Lawrence Berkeley National Laboratory (LBL), USA. After the shutdown of Bevalac in 1993, the QST-NIRS in Japan started the operation of Heavy-Ion Medical Accelerator in Chiba (HIMAC) in 1994. Although NIRS is a dedicated facility for heavy-ion radiotherapy, it can provide various ion beams for other experiments outside treatment hours. The available ions are He, C, Ar, Fe, and Xe with an energy maximum of 800 MeV/u (ion species dependent). There are five experimental beam lines at HIMAC, and a 3D field can be formed by the wobbler beam delivery system. In the past 25 years, many studies have been carried out, including those examining biological effects, radiation shielding, development and comparison of cosmic radiation detectors, and radiation tests of electric devices [48].

At the almost same time, the GSI Helmholtz Center for Heavy Ion Research (GSI) started the operation of SIS-18. GSI covers the wide research fields of nuclear physics, atomic physics, material science, plasma physics, biophysics, and clinical research, based on the heavy-ion accelerator complex. This facility can provide various ions from proton to uranium. The energy range is up to 2 GeV/u (ion species dependent) and is suitable to study the radiation effects of GCRs. Furthermore, the new FAIR accelerator complex is under construction at GSI. Heavy ions with energies up to 10 GeV/u will be available for radiation research in the near future [49].

The Brookhaven National Laboratory (BNL) in the USA has operated a very large heavy-ion accelerator complex, RHIC/AGS, for the study of nuclear and particle physics. In 2003, the NASA Space Radiation Laboratory (NSRL) was founded to study the health risks of cosmic radiation to crews. NSRL uses the BNL Booster synchrotron, which can provide ions from protons to gold, ranging in energy from 50 MeV/u to 2,500 MeV/u (ion species dependent) [50]. Although one of the difficulties of ground-based experiments is that cosmic radiation consists of a wide variety of ion species and energy ranges, NSRL produces a GCR simulated beam using rapid switching technology of ion species and ion energies.

In addition to these three research facilities, some institutes provide HZE beams for cosmic radiation research. In particular, 12 C-ion radiotherapy facilities are now in operation, including the Gunma University Heavy Ion Medical Center (GHMC) in Japan and the National Centre for Oncological Hadrontherapy (CNAO) in Italy. These facilities provide a C-ion beam from 100 MeV/u to 400 MeV/u, which is close to the GCR energy range. Researchers of cosmic radiation and the C-ion radiotherapy have common scientific interests in characterizing the biological effects of HZEs and contribute to this research field.

### 3.3. Microbeam Irradiation Facilities

Space radiation includes the HZE of GCRs, which is unlike radiation at ground level. Therefore, analysis of the hit effect of HZE is an important subject when evaluating space radiation risks for long-term manned missions.

HZE deposits concentrated energy along with its trajectory, and this manner of microdosimetric energy deposition of HZE is called the “ion track structure” [51]. The ion track structure is a characteristic of HZE and explains the difference between the biological effects of HZE and that of low-LET radiation. Because of this concentrated energy deposition of the ion track structure, a dose is deposited in close vicinity to the ion-hit position and does not extend further than a few micrometers away from this hit position. This results in a microscopic uneven dose distribution on radiation targets [52]. For example, when 1 Gy of HZEs with a LET higher than approximately 625 keV/*μ*m was used to irradiate a population of cells with nuclei with areas of 100 *μ*m^2^ using broad field beam irradiation, less than one ion hits the nucleus of a cell on average. Thus, a mixture of cells hit and not hit by an ion occurs. Even with low-LET radiation, when the number of hit events becomes small, a similar uneven spatial distribution of hit events will occur. However, the dose given to the cells with a single hit event is too small and not sufficient to induce cellular responses. In contrast, a single hit of HZE with high LET deposits a sufficient dose to cells that is biologically effective. Therefore, investigating the effects of HZE on a cell population with broad field beam irradiation faces two problems that arise from not uniformly irradiating the cell population. The first problem is that each cell in a cell population will not be hit with the same count of ions, making it difficult to evaluate the exact effect of a single-ion hit. The second problem is the radiation-induced bystander effect [53, 54], which is a phenomenon that ion-hit cells induce radiation responses on nonhit nearby cells by transferring the hit signal *via* biological pathways. This second issue contributes much more to the overall radiation effect than the situation of low-LET radiation.

A microbeam is an experimental method that targets and irradiates biological samples with a radiation spot on the micrometer scale under microscopic observation. Because a microbeam is able to irradiate each cell with a defined dose accurately, analysis of an evenly irradiated cell population is possible. Moreover, by irradiating only a part of the cell population, we are able to induce and analyze the radiation-induced bystander effect. Therefore, a microbeam is a useful approach to analyze biological effects caused by radiation having a microscopically nonuniform dose distribution like HZE. To analyze the hit effect of HZE, it is necessary to irradiate HZEs as a microbeam. There are many international facilities where biological targets can be irradiated with a microbeam [55–57]; however, most of them are limited to irradiating only protons and alpha particles. The sites that are able to irradiate microbeams of HZE are GSI [58], Munich University [59], Institute of Modern Physics in China [60], and QST-Takasaki [52, 61]. Of these four sites, three sites, except QST-Takasaki, are only capable of irradiating cultured cells. However, to evaluate the effects of HZE on human health, experiments using model animals are necessary. The heavy-ion microbeam at QST-Takasaki is able to irradiate cultured cells and small model animals with a HZE. Therefore, this facility has contributed to the analysis of radiation effects of HZE to cultured cells from the viewpoint of single-ion-hit effects [62] and bystander effects [63–65], as well as analyzed the effects of local HZE radiation on the whole body using the nematode *Caenorhabditis elegans* [66, 67] and Medaka fish [68]. Moreover, at QST-Takasaki, the development of a new microbeam beamline that generates a finer beam spot than the current system is taking place [69]. In summary, heavy-ion microbeams will contribute more in future research examining the effect of HZE radiation, which is a significant health risk for astronauts undertaking long-term projects in space.

## 4. Adverse Events Caused by Space Radiation

### 4.1. DNA Damage and Detection

Radiation exposure induces various biological effects with the main effect being damage to DNA. There are various types of radiation-induced DNA damage, including base damage, single-strand breaks (SSBs), and double-strand breaks (DSBs) [70–73]. Among them, DNA DSBs are the most severe DNA lesion. Therefore, organisms have various DNA damage repair pathways to ensure genome stability [70, 71, 73, 74]. However, if a large amount of damage occurs or the damage is not repaired correctly, cell death, cellular senescence, and tumorigenesis may be induced [71, 72, 75, 76].

The energy of radiation is important when considering radiation exposure in space. Radiation exposure on the ground is at low-LET radiation levels and includes X-rays and *γ*-rays, while GCR contains high-LET radiation such as energetic protons and heavy particle beams, i.e., HZE particles [77–79]. High-LET radiation exposure leads to dense ionization along their radiation tracks and induces complex DNA damage. These localized dense DNA regions of damage, within a few helical turns of DNA, are called “complex DNA damage (lesion)” or “clustered DNA damage (lesion)” and are difficult to repair when compared with that of normal DNA damage [80–83]. Therefore, even if the radiation dose is the same on the ground as that in space, the quality and amount of DNA damage that occurs will be different, and evaluating the quality and quantity of DNA damage induced by GCRs for precise assessment of the biological effects in space is required.

Although clustered DNA damage induced by high-LET radiation exposure is detected using agarose gel electrophoresis or the comet assay, the results are sometimes controversial because their sensitivity is limited [82, 84–88]. In recent years, several papers have reported visualization of DNA damage induced by high-LET radiation exposure using *γ*-H2AX, which is a marker of DNA DSB [17, 89–91]. These data have shown the different nature of DNA damage between low-LET radiation exposure and high-LET radiation exposure. Since *γ*-H2AX occurs in a DSB site-specific manner, it is used as a sensitive tool to detect DSB [92–94]. The Ohnishi group was the first to report that clear tracks of the *γ*-H2AX signal are detected in lymphoblastoid nuclei after spaceflight [17]. Additionally, a similar track of *γ*-H2AX was detected in fibroblast nuclei that had been cultured for 14 d at the ISS; however, these tracks were not observed for control samples on ground control samples [95]. Recently, we investigated DSB formation after exposure to different energy ion beams. Interestingly, our study indicated that the C-ion beam, which causes more complex DNA damage than the He-ion, induced larger *γ*-H2AX foci sizes than exposure to the He-ion beam. Both large and small sizes of foci formation were observed in C-ion and He-ion mixed beam irradiated cells (unpublished data). These results indicate that the radiation-induced *γ*-H2AX foci size depends on the energy of the radiation, suggesting that to correctly understand biological effects, not only the spatial formation of damage but also the size of damage needs to be considered.

Finally, radiation exposure in outer space occurs in a *μG* environment. Most studies conducted only analyze DNA damage caused by high-LET radiation exposure in a static environment, and it is unclear whether clustered DNA damage occurs and is repaired in a *μG* environment. Thus, to correctly understand the biological effects in outer space, it will be important to evaluate accurately the combined effects of *μG* and high-LET radiation exposure.

### 4.2. DNA Repair

As mentioned above, IR, including space radiation, generates various types of DNA damage. Among them, DNA DSB is the most serious damage, which can lead to tumorigenesis or cell death. Thus, organisms have developed DNA repair mechanisms to repair DSB damage. DSBs are mainly repaired by nonhomologous end-joining (NHEJ) and homologous recombination (HR) in eukaryotes [74]. Once DSB damages are generated following exposure to IR, the KU70/KU80 complex or MRE11/RAD50/NBS1 (MRN) complex is recruited to DSB damage sites. KU70/KU80 complex activates the NHEJ pathway with DNA-PKcs and the XRCC4/Lig4 complex, and these factors rejoin DSB ends. Since exposure of DNA to IR generates various forms of DSB ends, the resection of DSB ends by Artemis is essential for NHEJ progression. Such resection can lead to the loss of nucleotides and subsequent genomic instability. Hence, NHEJ is an error-prone repair system. Recruitment of the MRN complex activates the HR pathway, and this complex initiates the resection of the DSB ends with CtIP, followed by a longer resection with Exo1 or Dna2. As a result, more than 30 single-stranded DNA (ssDNA) tails are formed at both DSB ends. The replication protein A (RPA) complex then binds to ssDNA and is subsequently replaced with RAD51. Such ssDNA/RAD51 ends invade intact homologous DNA, and then error-free repair is completed using the intact homologous DNA as a template. Thus, as the HR mechanism needs an intact DNA template after replication, it is activated during the late S and G2 phase, whereas the NHEJ mechanism is activated at any point during the cell cycle [96]. NHEJ is used preferentially in higher eukaryotes such as humans. However, high-LET radiation such as heavy particle or *α*-rays, which is present in space radiation, generates various types of DNA damage (e.g., DSB, SSB, and oxidative damage) at the point of the irradiated areas. As NHEJ cannot repair such complicated DNA damage, HR is often activated for repair of this DNA damage in a cell cycle-independent manner [97]. However, cell cycle-independent use of HR, particularly in G1 may cause misrepair and subsequent genomic instability. Hence, RIF1 and 53BP1 can repress the unexpected activation of HR in G1 and function to select the correct repair pathway (i.e., NHEJ or HR) [96].

Acute exposure to 1 Gy of low-LET radiation such as a *γ*-ray could generate approximately 40 DSBs in a nucleus, ~1,000 SSB, and more than 1,000 base damages, as well as oxidation causing ~100,000 ionizations of various molecules in a nucleus simultaneously [98]. In the case of chronic irradiation by low-LET radiation, which is assumed to occur on space stations, the amount of DSB damage decreases to a negligible level. However, SSB and base damages remain and may represent a health risk. SSB damage and most types of base damage are repaired by base excision repair (BER), and cross-linked damage between adjacent bases such as a thymine dimer is repaired by nucleotide excision repair (NER) [99]. Some kinds of oxidative bases cause misinsertion of a base against the template DNA during DNA replication. Such misinserted bases are exchanged to correct bases by mismatch repair (MMR). Mistakes or incompletion of DNA repairs containing NHEJ and HR can lead to gene mutations and genomic instability, but the relationship with radiation carcinogenesis remains unclear.

### 4.3. Chromosomal Aberrations (CAs) and Micronuclei (MN)

CAs are cytogenetic biomarkers for exposure to IR and other DNA-damaging agents [100]. CAs can be measured using many types of cells including peripheral blood cells and are used frequently in epidemiological studies of humans, laboratory animals, and *in vitro* cell and tissue systems. The frequency of CAs in peripheral blood lymphocytes may be associated with the risk of human cancer [101].

CAs are classified into unstable and stable types [102]. Unstable types are unrepaired broken chromosomes and rearranged acentric, multicentric, or ring chromosomes. Unstable CAs are frequently lost with cell division because they are associated with impaired DNA replication of broken termini without telomeres or in chromosome segregation. Dicentric chromosomes are the most popular cytogenetic biomarker of unstable CAs. They can be identified easily with the conventional Giemsa staining because of their typical structure with two centromeres. Dicentric chromosomes are the biomarker of choice for investigating recent exposure to IR.

Stable CAs are rearranged monocentric chromosomes, which can be transmitted stably to daughter cells after cell division, and hence used as biomarkers of past exposures to IR. The conventional Giemsa staining cannot provide much information about stable CAs. The fluorescence *in situ* hybridization (FISH) technique with chromosome-specific DNA probes greatly improves the detection of stable CAs [103].

Chromosomes can be observed only in metaphase cells in their native forms. Premature chromosome condensation (PCC) techniques, which can induce condensation of chromosomes in cells at the interphase by fusion with mitotic cells or by chemical treatment, have improved CA analysis to detect DNA damage that has occurred in interphase cells [104, 105].

The MN assay is an alternative approach to detect DNA damage and used commonly because of its sensitivity, simplicity, and the speed by which cells can be scored [100]. MN are small pieces of DNA resulting from unrepaired DSBs or mitotic spindle damage that appears near the nucleus following cell division [106].

CAs in spacecraft crews have been analyzed since the 1960s to investigate genotoxic effects of space radiation and to estimate the received doses [107]. The frequency of total CAs seemed to be higher at postflight than at preflight, notably after flights longer than 180 days [107, 108]. However, the diversity of radiation history and personal susceptibility makes it difficult for epidemiological studies to estimate the risk of space radiation exposure. In addition, our knowledge of the effects of HZE particles involved in space radiation on induction of CAs is limited when compared with our understanding of low-LET IR. Studies using FISH painting revealed that HZE particles frequently induce highly complex chromosomal rearrangements when compared with the effect of low-LET IR [109]. Induction of mitotic CAs by HZE particles is complicated by their serious effects on cell cycle progression [110]. We recently compared induction of CAs and MN in C57BL/6J Jms mice at 1 and 2 months after exposure to several doses of X-rays (low-LET IR) or Fe-ions (HZE). FISH analysis of CAs in splenocytes showed that Fe particles are less effective at inducing translocations than X-rays when compared at the same physical dose. DNA DSBs induced by Fe-ions are probably not rejoined and mostly cause cell cycle arrest or cell death rather than result in induction of stable CAs [111]. Conversely, Fe-ions are more effective at inducing MN in bone marrow erythrocytes than X-rays, whereas the relative effectiveness of Fe-ions to X-rays was higher at a low dose (0.5 Gy) than that at a high dose (3.0 Gy) [112].

### 4.4. Genome Instability

Genomic instability refers to the accumulation of multiple changes within the genome of a cellular lineage to convert a stable genome to an unstable genome. Genomic instability is characterized by varied end points, for example, CAs, amplification of genetic material, micronucleus formation, and gene mutation.

Genomic instability can be induced by a high frequency of DNA damage [113] as DNA damages can cause inaccurate translesion synthesis past the damages or errors in repair, leading to mutation. IR can cause immediate effects such as mutation or cell death, observed within hours or a few days after irradiation. IR also induces delayed effects many cell generations after irradiation.

Genomic instability (delayed effect) caused by IR was first demonstrated by Kadhim et al. after alpha particle irradiation and indicated that many of the clonally derived cells that exhibited the unstable phenotype were not likely to have been traversed by an alpha particle [114]. IR is capable of inducing genome instability in mammalian cells, manifesting as delayed HR *in vitro* and *in vivo* [115, 116], which is detected in the progeny of an irradiated cell multiple generations after initial exposure. Genome instability is the driving force responsible for radio carcinogenesis, which can initiate cancer and augment progression [117–119].

Cosmic radiation contains proton, various HZE particle beams, and electron beams. As the heavy ion has a higher biological effect than proton or *γ*-rays, it is very important to study the effects *in vivo* and *in vitro*. For astronauts on space missions or people traveling in space, it is important to evaluate the risk of exposure to cosmic radiation, such as carcinogenesis.

**Figure 1:**
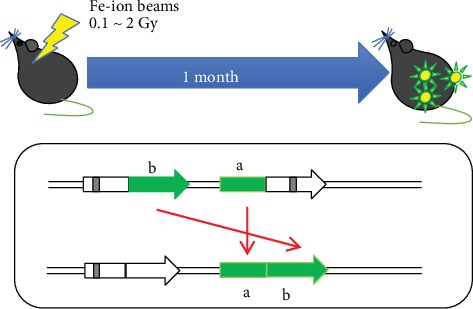
Wild-type EGFP (ab) fluorescence occurs as a result of HR between two *EGFP* genes (a and b) that are both inactive because of deletions (shadowed boxes).

DNA DSBs are repaired by the NHEJ and HR pathways. The correct balance of NHEJ and HR is essential for preventing genomic instability [120, 121]. HR is essential for repair of DSBs; however, too much HR activity can be detrimental and increase “genomic instability because HR carries the risk of misalignments that cause insertions, deletions, and a loss of heterozygosity (LOH) [122, 123]. However, there has been no observation of such genomic instability in animal tissues. In recent years, a research group at Massachusetts Institute of Technology established a model mouse system (RaDR mice) that enables evaluation of genomic instability using the green fluorescence of the green fluorescence protein (GFP) as an indicator [124]. In the mouse genome, a direct repeat HR substrate is targeted to the ubiquitously expressed Rosa26 locus and HR between two truncated enhanced GFP (EGFP) expression cassettes can yield a fluorescent signal (Figure 1).

Before using the mouse model, we used an *in vitro* system (RKO cells), namely a GFP direct repeat homologous recombination system. We demonstrated that DHR increases several-fold in response to low-LET X-rays and high-LET C-ion radiation [116, 125].

Using the RaDR model mouse, we confirmed that the HR frequency is related to thymic lymphomas. When 5 weeks old, RaDR mice were irradiated with 1.8 Gy *γ*-rays per week for 4 weeks (total dose 7.2 Gy), and about half of the individuals developed thymic lymphoma by 150 days. Our results indicated that a significant increase in GFP-positive cells was observed in infiltrated lymphoma. Two months after the irradiation, the frequency of GFP-positive nucleated cells (HR frequency) increased in the thymus, bone marrow, and spleen. In contrast, when model mice were irradiated with 0.5 Gy Fe-ion beam, the HR frequency in bone marrow or spleen cells was observed to increase significantly. Additionally, we found that the HR frequency significantly decreased under a radioadaptive response- (RAR-) inducible condition when compared with that under a non-RAR-inducible condition [126].

### 4.5. Carcinogenesis

Carcinogenesis is a major concern for future space missions, especially space missions that will be for long durations [127–129] because astronauts will be constantly exposed to IR from natural radiation sources. The radiation field in space contains electrons, protons, alpha particles, and heavier ions up to HZE-charged particles. In addition, inside spacecraft, various secondary radiations including neutrons are created by interactions between primary radiation and materials of the spacecraft.

The carcinogenic potential after radiation exposure has been revealed by epidemiological data from atomic bomb survivors [130]. However, there is insufficient data delineating the carcinogenic potential of HZE-charged particle radiation. Therefore, estimation of the cancer risk after exposure to each HZE particle or neutron using animal experiments is important. RBE values are given as the ratio of the absorbed doses of two types of radiation producing the same specified biological effect under identical irradiation conditions. Cucinotta et al. [131] used RBE values from various animal experiments for predicting the risk of cancer after exposure to HZE and fission neutrons, and the used RBE values were 2 to 10 and 4 to 20, respectively. Imaoka et al. [132] summarized RBE of the risk of cancer after exposure to protons, C-ions, or neutrons to estimate secondary cancer after radiation therapy. The RBE was less than 2 for protons and less than 20 for C-ions and neutrons. These animal data revealed that RBE values are variable for tissues type, radiation types, and age at the time of irradiation.

The greater carcinogenesis effects of HZE particle radiation have been analyzed from the viewpoint of a targeted effect (genetic change) and nontargeted effects. C-ion-induced lymphomas showed a marginal increase in the frequency of large interstitial deletions at various sites across the genome when compared with that of photon-induced lymphomas [133]. HZE particle irradiation promoted more aggressive cancers, such as increased growth rate, transcriptomic signatures, and metastasis when using a radiation/genetic mammary chimera mouse model of breast cancer [134]. This suggests that the nontargeting effects of HZE particles were more effective than the reference *γ*-radiation. Unfortunately, there is still a paucity of data on this subject.

Considering radiation exposure in deep space, the health risk of exposure at low dose and low-dose-rate radiation from GCR is also important. Chronic exposure to *γ*-rays or X-rays has been reported to reduce dramatically the risk of carcinogenesis when compared with that of acute exposure [135, 136]. Therefore, cancer risks after exposure to low-dose-rate HZE require further clarification. In future experiments, more animal data are required to determine the RBE of cancer risk after exposure to HZE particles or neutrons.

### 4.6. Central Nervous System (CNS) Response

In the last 10–20 years, risk assessment of space radiation has focused on the risks of cancer. In addition to the risk of cancer, NASA recently began focusing on the risks to the CNS. The CNS consists of the brain and spinal cord. The brain is the body's most complex organ and its spatial architecture. There are approximately 86 billion neurons and glia cells of the about the same number in the human brain [137, 138], all of which communicate to form circuits and share information. It is therefore very difficult to evaluate the radiation risk to the brain. Thus, it is necessary to evaluate the response of individual cells in the brain directly to radiation as a simple, accessible model.

The brain is a largely radioresistant organ [139]. However, ground-based animal studies indicate that space radiation alters neuronal tissue and neuronal functions such as excitability, synaptic transmission, and plasticity. HZE particles have been demonstrated to inhibit neuronal connectivity, neuronal proliferation, and neuronal differentiation and to change glial characterization [140]. We summarize the current knowledge of neuronal and glial responses caused by HZE irradiation less than 2 Gy (Table 1).

Thus, many researchers observed the response of the brain to radiation using short-term, higher-dose-rate exposures of radiation, which does not accurately reflect the conditions in space. The long-term effects of these doses of radiation on the CNS are largely unknown. Acharya et al. exposed mice to chronic, low-dose-rate (1 mGy/day) radiation for 6 months to investigate how deep space travel could affect the CNS [152]. They found that the radiation exposure impaired cellular signaling in the hippocampus, a part tied to learning and memory, and the prefrontal cortex, which plays a role in higher cognitive functions, resulting in learning and memory impairments. They predict that during a deep space mission, 1 in every 5.1 astronauts would experience anxiety-like behavior, and 1 in every 2.8 astronauts would experience certain levels of memory impairments. These results suggest that chronic, low-dose-rate radiation exposure from deep space travel may pose considerable risks for cognitive performance and health. For the assessment and management of human health in space, it is necessary to obtain more basic data of the effects of radiation on the brain. Additionally, it is important for us to progress with the developments of methods and protective materials that shield radiation effects.

### 4.7. Motility Disturbance

Adverse effects of high-LET radiation, an important component of cosmic rays, on the functions of biological systems are a potential risk in interplanetary manned space missions. Therefore, analysis of the effects of high-LET radiation on animals at an individual level and focusing on the impact of such radiation on biological functions are important for space missions. The effects of high-LET radiation exposure on several behaviors including muscle movements have been investigated using the nematode *C. elegans* [66, 67, 69, 153, 154], which is an experimental model organism and a powerful tool to study the effects of radiation. In this animal, locomotion, including forward and backward movements and turns, is carried out by 95 body wall muscle cells, for which the fate of each cell from its birth to death can be easily determined. Locomotion (motility) of adult *C. elegans* on an agar plate without food was reported to decrease in a dose-dependent manner immediately after whole-body irradiation was administered using both high-LET radiation (^12^C, 18.3 MeV/u, LET = 113 keV/*μ*m) [67, 153] and low-LET radiation (^60^Co *γ*-rays) [155]. The RBE ratio of high-LET radiation relative to low-LET radiation for inhibition of locomotion was 1.4 [153]. If the radiation effects were mainly caused by DNA damage, it is generally thought that the effects of high-LET radiation would be several times higher than those of low-LET radiation. Therefore, the reduction of motility in *C. elegans* following exposure to high-LET radiation is not caused by DNA damage and is likely induced by another factor. Recovery of motility shortly after irradiation supports the hypothesis that DNA damage is not responsible for IR-induced reduction of motility. In particular, an important factor that induces radiation effects is reactive oxygen species (ROS) produced by IR. Exposure to IR results in the formation of free radicals such as OH• or H•, and the reactions of free radicals cause the production of ROS, including hydrogen peroxide (H_2_O_2_). Experimental results showed that *C. elegans* motility was H_2_O_2_ dose-dependent, indicating that radiation-induced reduction in motility is caused by IR-produced H_2_O_2_ [155]. Moreover, the results of region-specific irradiation showed that motility was not reduced significantly by irradiation of any of the individual tissues in a ∅ 20 *μ*m region, including the CNS, intestines, and tail. This suggests that radiation reduces locomotion by a whole-body mechanism, potentially involving motor neurons and/or body wall muscle cells, rather than affecting motor control *via* the CNS and the stimulation response [67].

In studies of stress responses, disturbances to muscle cells induced by various stresses and stimulations have been well investigated. Wang et al. showed that mitochondrial dysfunction is related to muscle atrophy [156], and extracellular matrix (ECM) stability is necessary for maintaining muscle health. In addition, Momma et al. investigated alterations of Ca^2+^ homeostasis and mitochondrial morphology *in vivo* in body wall muscles of *C. elegans* exposed to an elevated temperature. The results showed that heat stress for 3 h at 35°C increased the concentration of free Ca^2+^ and led to mitochondrial fragmentation and subsequent dysfunction of the muscle cells [157]. Furthermore, it was reported that mitochondrial dysfunction acts as an intramuscular signal that, *via* excessive Ca^2+^ release, activates ECM-degrading enzymes to reduce ECM content and, subsequently, results in the structural and functional decline of muscles [158].

Although reduction in motility of body wall muscles recovers within several hours after whole-body irradiation with less than 1,000 Gy of high-LET radiation and the effects are masked, the disturbance observed after whole-body irradiation with more than 1,000 Gy of high-LET radiation might be induced by the above mitochondrial mechanisms. Further studies that focus on the effects of radiation to the homeostasis of muscle cells are required.

### 4.8. Visualization of Adverse Events

The cellular response to DNA damage varies according to the cell type, the stage of the cell cycle, and extent of damage [159]. More than 50 years have passed since the first observation of cell cycle-dependent DNA damage was made by using synchronized HeLa cell populations [160, 161]. These classical studies concluded that mitotic cells are hypersensitive to X-ray irradiation, which inactivates the DNA DSB repair pathway. Cell survival was maximal when cells were irradiated during the early postmitotic (early G1) and premitotic (S to G2) phases and was minimal during the mitotic (M) and late G1 or early DNA synthesis (early S) phases. However, the conventional “arrest-and-release” methods using pharmacological reagents or the mitotic shake-off method cause more or less adverse cellular perturbations and do not ensure complete cell cycle synchronization of tumor cells.

**Figure 2:**
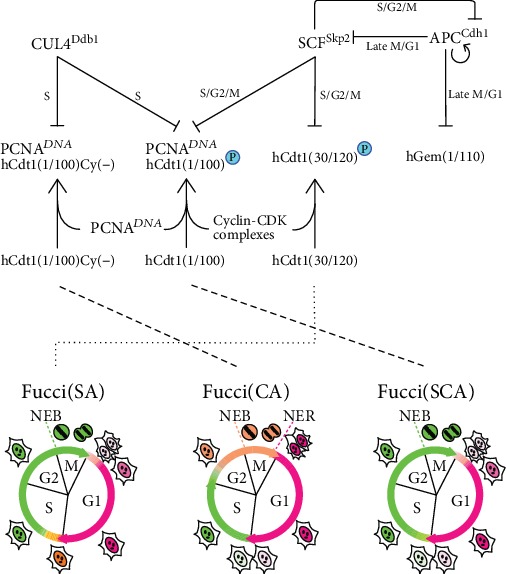
Cell cycle-phasing capabilities of the Fucci technology. Cell cycle regulations involving E3 ligase activities of CUL4^Ddb1^, SCF^Skp2^, and APC^Cdh1^. Molecules whose intracellular concentrations or enzymatic activities change in a cell cycle-dependent manner are shown in color. PCNA*^DNA^*: DNA-bound PCNA. Data adapted from Sakaue-Sawano et al. [164].

Recently, a variety of fluorescent protein- (FP-) based methods for visualizing cell cycle progression at the single cell level have been developed, enabling researchers to analyze cell cycle progression without affecting normal cellular functions. Fucci (fluorescent ubiquitination-based cell cycle indicator) harnesses the cell cycle-dependent proteolysis of Cdt1 and Geminin. Fucci highlights the cell cycle transition from G1 to S phase with high color contrast, like a traffic signal: red and green mean “stop” and “go,” respectively, for the transition (Figure 2) [162, 163]. SCF^Skp2^ and APC^Cdh1^ E3 ligases are involved in the degradation of Cdt1 and Geminin, respectively. Over the course of the cell cycle, these two E3 ligase activities oscillate reciprocally and the protein levels of their direct substrates oscillate accordingly. To label S–G2–M-phase nuclei green, the Fucci probe has a green-emitting FP fused to the APC^Cdh1^-mediated ubiquitylation domain (1–110) of human Geminin (hGem) (Fucci-S/G2/M-Green); this chimeric protein is the direct substrate of APC^Cdh1^ E3 ligase. To label G1-phase nuclei red, the probe has a red-emitting FP fused to residues 30–120 of human Cdt1 (hCdt1) (Fucci-G1-Red); it contains the Cy motif (amino acids 68–70), which binds to the SCF^Skp2^ E3 ligase. The combination of the RFP-labeled hCdt1(30/120) and GFP-labeled hGem(1/110) can be called Fucci(SA) because they monitor the balance between SCF^Skp2^ and APC^Cdh1^ E3 ligase activities.

Eukaryotic cells spend most of their life in interphase of the cell cycle. Understanding the rich diversity of genomic regulation that occurs in interphase requires the demarcation of precise phase boundaries *in situ*. Although Fucci(SA) highlights the G1/S phase transition with yellow fluorescence, it does not provide a fluorescent readout for distinct interphase boundaries. Additionally, Fucci(SA) has a fluorescence gap in very early G1 phase, making it difficult to continuously track cell positions in all phases of the cell cycle.

In 2017, we engineered the hCdt1-based probe to be sensitive to CUL4^Ddb1^ in addition to or instead of SCF^Skp2^ [164]. As the PIP box (amino acids 1–10 of hCdt1) is a specific substrate of CUL4^Ddb1^, hCdt1(1/100), which retains both the PIP box and Cy motif, is targeted by both SCF^Skp2^ and CUL4^Ddb1^. We also constructed hCdt1(1/100)Cy(–), which is a specific substrate of CUL4^Ddb1^. By combining hCdt1(1/100)- and hCdt1(1/100)Cy(–)-containing red-emitting probes with hGem(1/110)-containing green/yellow-emitting probes, we developed Fucci(SCA) and Fucci(CA) probes, respectively, which have increased the versatility of the Fucci technology for new biological studies of cell cycle interphase regulation. Although Fucci(CA) monitors the balance between CUL4^Ddb1^ and APC^Cdh1^ E3 ligase activities, Fucci(CA) can distinguish clear interphase boundaries between G1, S, and G2 phases.

We have demonstrated that Fucci(CA) can be used to

1. fully highlight the short G1 phase of rapidly proliferating mESCs
2. continuously track cell positions in all phases of the cell cycle
3. detect cell cycle- (S phase) specific sensitivity (HeLa cells) to UV irradiation
4. explore cell cycle-specific intracellular signaling
5. visualize a cell cycle-specific response or homeostatic balance to space radiation

To investigate the impact of space radiation and *μG* on “Living in Space,” a variety of FP-based approaches had been launched. Harada et al. introduced an EGFP-53BP1M FP probe to visualize the diversity of the radiation-induced DNA damage responses in real time [165]. Ishii's group reported that B16BL6 cells in the early S phase were the most susceptible to radiotherapy [166]. Live imaging technology using FPs is expected to make significant contributions to the direct visualization and detailed understanding of radiation adverse events.

## 5. Combined Biological Effects

### 5.1. Radiation and *μG*

The biological effects of radiation and *μG* in space experiments are summarized in Table 2. In a previous short mission, there was no appreciable difference in results between space and ground samples because exposure to space radiation occurred at a low dose. Therefore, various living systems have been irradiated before spaceflight to clarify the effect of *μG* on the radiation-induced DNA damage response, but there was no appreciable difference in results [167–171]. However, synergistic effects between radiation and *μG* have been reported [172–177], and they can suppress each other's effects [172, 178]. There is the still no consensus on whether radiation and *μG* have combined effects [179, 180]. JAXA developed not only the Cell Biology Equipment Facility (CBEF) [181] but also a mouse habitat unit cage (MHU) [182], which provides long-term artificial gravity for control experiments in space. This experimental platform provides the opportunity to investigate the specific impacts of space radiation and *μG* for future human space exploration [181].

The biological effects of radiation and simulated *μG* in ground experiments are summarized in Table 3. To clarify the effects of *μG* at ground level, researchers have used rotating devices, such as a rotating wall vessel bioreactor (RWV; Synthecone, Houston, TX, USA) and the random positioning machine (RPM; Dutch Space, Netherlands), which are pieces of equipment that continuously rotate a sample. These devices can equalize the gravity vector and cancel the effect of gravity, thereby simulating *μG*. However, there are two major limitations associated with this approach: (i) it is necessary to stop rotation during irradiation as the sample was exposed to radiation outside the incubator after or before rotation with a RWV [183–188] and (ii) nonuniformity of dose flatness in the irradiation area occurs because of chronical irradiation of a rotating sample with a RPM [189, 190]. To address these problems, we have developed a system of simultaneous irradiation in simulated-*μG* (SSS) using 3D clinostat [191, 192]. Our SSS is based on technologies related to X-ray irradiation with a high-speed shutter [191] and C-ion radiotherapy such as accelerator systems and respiratory gating systems [192].

Using this SSS, we reported that simultaneous exposure of human fibroblasts to simulated *μG* and radiation results in a greater frequency of chromosomal aberration than in cells exposed to radiation alone [193]. The expression of cell cycle-suppressing genes decreased and that of cell cycle-promoting genes increased after C-ion irradiation under simulated *μG* [194]. Assessment of the cancer risk associated with space radiation in the conventional manner based on data of radiation quality and quantity from cells irradiated under static conditions might underestimate the potential risk to astronauts. Nonetheless, examination of endpoints and *in vivo* model systems under the combined effects of radiation and *μG* are required.

In the near future, there is also a need to investigate the biological effect of partial gravity such as 1/6*G* and 3/8*G* on the response to radiation for manned missions to the Moon and Mars. Two simulated partial gravity devices using the RPM, one by applying specific software protocols to drive the RPM motors and the other involving integrating a centrifuge into the RPM, should become useful tools [195]. The actual effects should be tested either in a proper centrifuge experiment on the ISS, such as CBEF [181] and MHU [182], or actually on the surfaces of the Moon and Mars.

### 5.2. Combined Effects of *μG* and UV Radiation on Plants

Plants supply nutrients and oxygen to humans under a resource-recycling system on Earth and also in space. All organisms, including plants, have evolved protection mechanisms against environmental stresses. However, the environment in space differs dramatically from that on Earth. Can all organisms adapt to the environment in space and live healthy? In addition, there is the possibility that the higher intensity of UV radiation, which is a driving force of evolution, and the complex cosmic IR in space could lead to an increase in the mutation frequency. Currently, *μG* has been reported to cause cellular oxidative stress that leads to production of ROS and endoplasmic reticulum stress in experimental animals [196–198]. In addition, Sugimoto et al. reported that the environment during spaceflight induces oxidative stress and ROS gene network activation in the space-grown Mizuna plant [199]. The mechanisms by which *μG* elicits these cellular responses remain poorly understood, although very interesting results have been reported recently. For example, simulated *μG* induces autophagy *via* mitochondrial dysfunction in human Hodgkin's lymphoma cells [196] and TCam-2 cells [197]. The results of proteomic and metabolomics analysis of human primary osteoblasts exposed to simulated *μG* suggest that *μG* suppresses bone cell function, impairing mitochondrial energy potential and the energy state of the cell [200].

**Figure 3:**
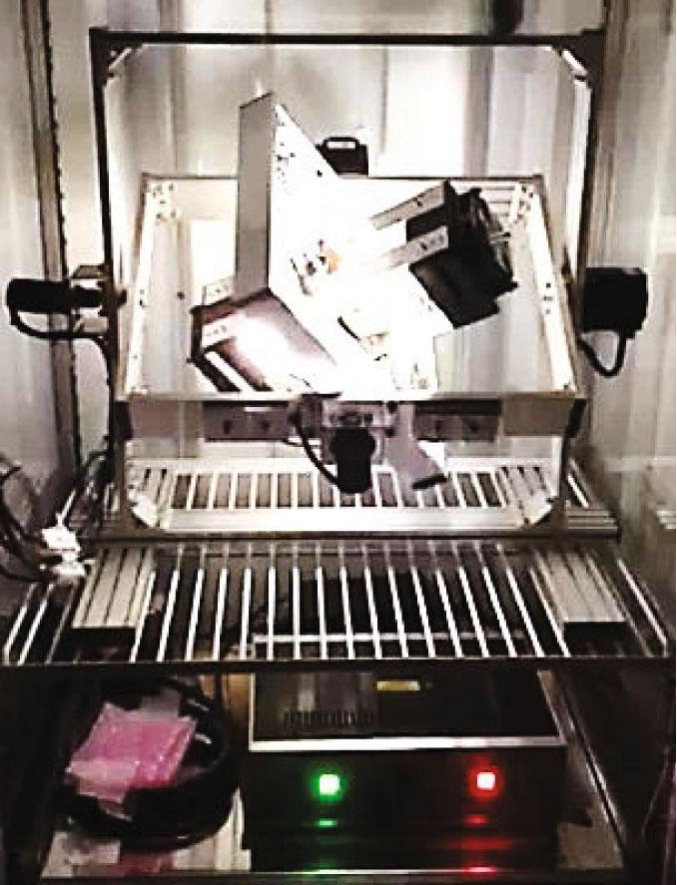
A 3D clinostat quipped with a UV-visible light unit. The UV-visible light unit is composed of white and UV-B- (280 nm) light-emitting diodes (LEDs).

To plan cultivation of plants in space including Mars, we need to identify what plants to use and whether to use sunlight or an artificial light source for growth. Negative effects of UV radiation can be avoided if plants are grown under artificial light without sunlight. However, we need to address some issues. For example, (1) it is difficult to grow plants uniformly in the same growth chamber, because the optimal wavelength and light intensity differ for different vegetable plants; and (2) growing plants in a growth chamber under artificial light is very costly because of the consumption of electric power. Conversely, if plants are grown using sunlight, the potential negative effects from UV radiation are unavoidable. It is unclear whether various UV-B protection mechanisms, which have evolved under 1*G*, would function properly under lower gravity. It is thus important to investigate the potential ability of plants to adapt to the environment of space. For this purpose, utilization of facilities on orbiting space platforms such as the ISS is essential, although we cannot repeatedly and frequently conduct experiments on the ISS. To disturb the gravity direction or produce simulated *μG* on the ground, a 3D clinostat is useful and convenient (Figure 3).

Therefore, it is necessary to understand the combined environmental effects of space on plants at the molecular, cellular, and whole-plant levels and understand not only the transient, short-term (one generation) effects but also long-term (next, subsequent generations) effects under space environmental conditions through space experiments or experiments using equipment such as a 3D clinostat. Such experiments clarify direct and/or indirect gravity effects on vegetative and reproductive growth, provide new evidence of antigravity reactions, and possibly find not only novel biological knowledge, such as molecular mechanisms in gravity reactions, but also novel growth controls in crop production on Earth. In addition, experiments that include the space-specific radiation environment will help elucidate the combined influence of low gravity and high-level visible UV and space radiations on plant growth and regeneration in a whole growth stage. However, a study about such combined effects on organisms as well as plants has only recently been initiated. Such studies, both on the ground and on orbiting space platforms such as the ISS, should be promoted to establish sustainable life support systems for securing long-term human life in space and on the Moon and Mars.

### 5.3. Radiation and Stress

Stress refers to conditions where an environmental demand exceeds the natural regulatory capacity of an organism, in particular situations that include unpredictability and uncontrollability, and psychological stress is one of two basic kinds of stress [201]. Psychological stress and radiation are known to cause various adverse effects on humans. Radiation is a carcinogen, and long-lasting psychological stress may affect the overall health and ability to cope with cancer. Whether psychological stress influences susceptibility to radiation, radiocarcinogenesis in particular, is of great concern for both academia and the public [202]. Using a laboratory mouse model for chronic restraint-induced psychological stress, the pioneering work on concurrent exposure of *Trp53* heterozygous C57BL/6 mice to psychological stress and total body *γ*-rays showed that psychological stress modulates susceptibility to radiation, causing increased susceptibility to radiocarcinogenesis in *Trp53*-heterozygous mice underlying the mechanism of *Trp53* function attenuation [203]. In recent years, studies using the same chronic restraint system, wild-type C57BL/6J male mice aged 5 weeks and total body exposure to 4 Gy X-rays, showed that psychological stress has minimal modifying effects on radiation-induced hematopoietic toxicity and genotoxicity measured as a peripheral blood histogram, MN in the erythrocytes of bone marrow, and splenocyte CAs (insertions, dicentrics, and fragments), suggesting that chronic restraint-induced psychological stress does not appear to synergize with the clastogenicity of low-LET radiation in wild-type animals [204, 205]. Interestingly, in the animal model for psychosocial stress using 6- or 8-week-old male ddY mice (model mouse for spontaneous IgA nephropathy) and SAMP10 mice (model mouse for accelerated senescence), results of concurrent exposure to both psychosocial stress and X-rays at a dose of 3–6 Gy showed increased acute damage, namely, reduced 30-day survival, and decreased erythrocyte and leukocyte counts in the peripheral blood and hypocellular bone marrow, indicating psychological stress promotes radiosensitivity of bone marrow in these particular mice [206]. Interestingly, investigation using the mouse model for chronic restraint-induced psychological stress, *Trp53* heterozygous C57BL/6N male mice aged 6 weeks, and high-LET Fe particle irradiation at 0.1 or 2 Gy showed that concurrent exposure to psychological stress and 0.1 Gy Fe irradiation resulted in increased hematopoietic toxicity and genotoxicity measured as MN in the erythrocytes of the bone marrow and splenocyte CAs [207–209]. In contrast, in the mouse testis, concurrent exposure to 0.1 Gy Fe irradiation did not induce any increased apoptosis and autophagy inhibition [210]. These results indicate that psychological stress does not exacerbate radiation effects. These findings also suggest that studies on concurrent exposure should be performed using different endpoints in different tissues in both short- and long-term models for chronic restraint-induced psychological stress. In summary, concurrent exposure of wild-type mice to psychological stress and low-LET radiation did not suggest an additive effect for induction of hematopoietic toxicity and genotoxicity but promoted radiosensitivity of the bone marrow in some disease-prone mice. In contrast, concurrent exposure of *Trp53* heterozygous mice to psychological stress and high-LET radiation suggested an additive effect for induction of hematopoietic toxicity and genotoxicity. To reduce health risks from exposure to radiation by active intervention, further investigations are needed to collect more data that provide insights into the mechanisms underlying the alterations in susceptibility due to psychological stress modulation.

## 6. Radiation Exposure Management

To prevent IR-induced carcinogenesis, the exposure dose in spaceflight is limited to a level that will not result in exposure-induced death (REID) from fatal cancer over a career of more than 3%, at the 95% upper confidence interval of the risk calculation [129]. Based on this concept, missions in space are currently planned to last less than 180 days [211]. However, it has been suggested that there are individual differences in the IR-induced cancer risk within human populations [212]. Three factors that underlie individual IR-induced cancer risk, i.e., age, sex, and smoking, are already considered when determining the safe number of days in spaceflight [213]. Here, we review genetic variants in the DNA repair genes as an important factor underlying the individual differences in IR-induced cancer risk.

In human cells, DNA repair systems monitor and repair DNA DSBs to maintain genomic integrity. If IR-induced DSBs are left unrepaired, they can alter the information stored in the genome to cause carcinogenesis. It is thus useful to measure the capacity of cells to repair DSBs to understand how prone individuals are to IR-induced carcinogenesis. The cytokinesis-blocked MN assay, a procedure established to evaluate the capacity of cells to repair DSBs by counting MN derived from unrepaired DSB-induced chromosomal fragments, has revealed the existence of cases in which the capacity to repair DSBs has been slightly decreased by IR within healthy individuals and those with breast cancer [214]. The FISH painting analysis, which monitors IR-induced unstable ring and multicentric chromosomes, also demonstrated the heterogeneity of the capacity to repair DSBs after IR within human populations [215]. Interestingly, genome-wide association studies (GWASs) have revealed that many nucleotide variants in DNA repair genes are linked to an enhanced risk of cancer in normal individuals [216]. These findings in the fields of radiation biology and epidemiology have suggested that the personalized risk of cancer after IR exposure might be attributable to variants in DNA repair genes.

To clarify whether variants in DNA repair genes are involved in the risk of IR-induced cancer, it is informative to compare chromosomal instability after IR exposure of primary cells with or without the variant of interest, such as skin fibroblasts and peripheral blood lymphocytes. However, the capacity of primary cells to repair DSBs is affected by the diverse genetic backgrounds within human populations [217]. It is therefore essential to evaluate the effects of candidate variants on the capacity of cells to repair DSBs in a uniform genetic background. Genome-editing technology is beneficial in this regard because it enables the introduction of candidate variants into human cultured cells with a uniform genetic background. Comparison of IR-induced chromosomal abnormalities in genome-edited cells can then reveal whether a candidate variant is able to repair DSBs within human populations. Previously, we used this approach to demonstrate that ataxia-telangiectasia mutated (*ATM*) heterozygous mutations, which are present at a rate of around 1% in human populations, are indeed associated with the individual capacity of cells to repair DSBs [217]. Besides *ATM* gene mutations, germline mutations of DNA repair genes in human populations have been reported, such as *MRE11A*, *NBS1*, *Rad50*, *Artemis*, and *DNA Lig-IV* [212]. These mutations are generally rare, while heterozygous *BRCA1* and *BRCA2* mutations for hereditary breast and ovarian cancers are estimated to be present at a rate of 0.05–1% in human populations [218, 219]. The extent to which these mutations contribute to the capacity to repair DSBs remains unclear but should be resolved to achieve personalized radiation exposure management. Further studies using an approach combining the fields of epidemiology and functional genomics are needed to understand the genetic basis of individual differences in IR-induced cancer risk.

## 7. Protection from Radiation

### 7.1. Protective Agents

Many biological effects such as cell death and inflammatory responses due to radiation exposure are caused by DNA damage [71, 72, 75, 76]. Therefore, various drugs that aimed at decreasing induced DNA damage have been studied as radioprotective agents. Radiation induces DNA damage both directly and indirectly through radicals generated in response to intracellular water molecules. Thus, there are numerous studies evaluating antioxidants that suppress radiation-induced radical generation [220–224]. In particular, the effects of vitamin C and vitamin E have been studied for many years as antioxidants with radioprotective effects. Our group has assessed radioprotective effects of ascorbic acid (AA) to patients before cardiac catheterization (CC) for diagnostic purposes. Although we did not find satisfactory evidence to show that AA treatment reduces *γ*-H2AX foci formation immediately after CC, a slight decrease in DNA damage in the group of AA treatment was detected [225]. However, the results vary depending on the animal model used, the radiation dose, and the method for evaluating the protective effect [226–230]. In addition to vitamins C and E, radioprotective effects of nitroxide compounds as strong radical scavengers have also been analyzed [220, 231–233].

Currently developed radioprotective drugs are unsuitable as radioprotectants in outer space because the situation of radiation exposure differs to that of previous ideas. In outer space, suitable radioprotective drugs should protect against chronic exposure to low dose and a low dose rate of high-LET radiation, and not the acute high-dose radiation exposure found in radiotherapy. Drugs suitable for humans living in space must treat both unexpected high-dose radiation exposure due to solar flares and the suppression of DNA damage by space radiation that occurs constantly. Therefore, it is necessary to validate a radioprotective drug that can be taken daily with minimal side effects. For this purpose, it may be effective to develop functional space foods with a radioprotective effect that can be ingested continuously in outer space [234, 235]. Currently, our group is examining the radioprotective effect of piceatannol, which is an ingredient of passion fruit and displays strong antioxidant activity. We have confirmed that suppression of DSB after not only low-LET radiation exposure but also various high-LET radiation exposures occurs when using piceatannol (unpublished data). The development of various radiation protection agents is expected to progress in the future. We emphasize that there is a need for the development of protective agents against not just space radiation but also various space environmental risks.

### 7.2. Historical Overview and Perspective of Basic Research for the Development of Biological Strategies

Unfortunately, the development of a biological strategy for protection of our body from space radiation has not been achieved. To accomplish this, a basic knowledge about adverse effects of space radiation toward human health is required. In particular, we need to understand the radiosensitivity of each tissue. A French oncologist, Jean Alban Bergonié, and a French dermatologist, Louis Tribondeau, worked together between 1904 and 1906 and formulated a fundamental law in the field of radiation biology regarding the difference in radiosensitivity of normal tissues. They observed damage in the testis of male rats under a microscope after whole -body X-ray irradiation and found that biological effects of radiation were severer in the order of spermatogonia, spermatocyte, spermatid, and sperm. They generalized the result and formulated the so-called the Law of Bergonié and Tribondeau, which theorized that radiation causes severer damage to a tissue (1) when reproductive activity of cells in the tissue is greater, (2) when the karyokinetic fate of cells is longer (in other words, when the length of time that cells proliferate actively is longer), and (3) when morphology and function of cells are less differentiated. Based on this, radiosensitivity of representative tissues is classified as a summary in Table 4.

Accumulating evidence has demonstrated that the law certainly applies to many tissues; however, there are some exceptions. For example, Regaud claimed that spermatogonia in young rats are less radiosensitive than those in adults, though their proliferation rates are similar [236]. Using tobacco leaves, whose cell division rate significantly decreases as they grow, Haber and Rothstein demonstrated that radiosensitivity was almost the same between dividing and nondividing tissues [237]. Meyn and Jenkins measured the efficiency of DNA strand break formation in normal tissues of mice after whole-body irradiation and found that the least breaks were produced in the gut when compared with those of other tissues such as the bone marrow, spleen, brain, kidney, testis, and liver [238]. Ueno et al. recently found that quiescent melanocyte stem cells (McSCs) were more radiosensitive than coexisting nonquiescent McSCs and suggested that tissue radiosensitivity depends on the state of somatic stem cells under their microenvironment [239]. The law of Bergonié and Tribondeau needs to be revisited to integrate current knowledge about differences in radiosensitivity between various tissues.

Adverse effects of space radiation have been investigated under the various limitations of experimental settings; there is no way to separately evaluate radiosensitivity of each tissue using acute and monoenergetic beams [240]. To conduct more integrated analyses, our efforts in establishing a platform for *in vivo* animal studies are required. We can then analyze the effect of multiple factors (including low gravity and tissue microenvironment) on the efficiency of repair of DNA damage caused by space radiation. In particular, *in vivo* research using imaging techniques or genetically modified animals should provide spatiotemporal information about these factors. Moreover, we should conduct this research under various kinds of radiation that mimic space radiation with complex energy spectra and diverse ionic compositions. These approaches are expected to give us important information about radiosensitive tissues that should be protected from space radiation during ultralong spaceflights. Additionally, these approaches may lead to the development of radioprotective agents and also a system to select an astronaut who is potentially radioresistant.

## 8. Radioresistant Organisms

Organisms on Earth are protected from harmful space radiation by the electromagnetic field of our planet, most organisms including us are vulnerable to radiation, and radiation damage is one of the most severe risks to human health in long-term space flights. Some species on our planet, however, exhibit extraordinary resistance against high doses of radiation. Elucidating the molecular machinery responsible for these extraordinary radioresistance may aid the development of novel technologies that alleviate biological damage caused by radiation.

Most of the well-known radioresistant organisms are single-cellular prokaryotic organisms, such as archaea and bacteria. *Deinococcus radiodurans*, one of the most famous radioresistant bacteria, is reported to survive without loss of viability even after irradiation with 5,000 Gy of *γ*-rays [241, 242]. Although the genome DNA of *D. radiodurans* is heavily fragmented by high-dose irradiation, the DNA fragments are rapidly repaired to a complete circular genome by extensive DNA repair processes likely using their polyploid genome [243]. Mutation in the DNA repair pathways drastically compromises the radioresistance of *D. radiodurans*, suggesting that DNA is the most vulnerable target to radiation, and the powerful DNA repair system plays important roles in the high radioresistant capacity of this bacterium [243]. In addition to unicellular organisms, some animals such as tardigrades, bdelloid rotifers, and a sleeping chironomid, also exhibit exceptional tolerance against high doses of irradiation [244–248]. Intriguingly, these radioresistant animals also exhibit tolerance against almost complete dehydration. In a dehydrated state also known as “anhydrobiosis,” they can withstand several thousand Gy of *γ*-irradiation. Some tardigrade species and a sleeping chironomid were reported to survive direct exposure to space in a desiccated state, suggesting that they are resistant even against space radiation [249, 250]. Because biological damage by radiation, e.g., DNA lesions, partly overlaps with damage caused by desiccation, similar resistance machinery may be used to mitigate these two stressors, and coevolution of radioresistance and desiccation tolerance has been proposed [246].

**Figure 4:**
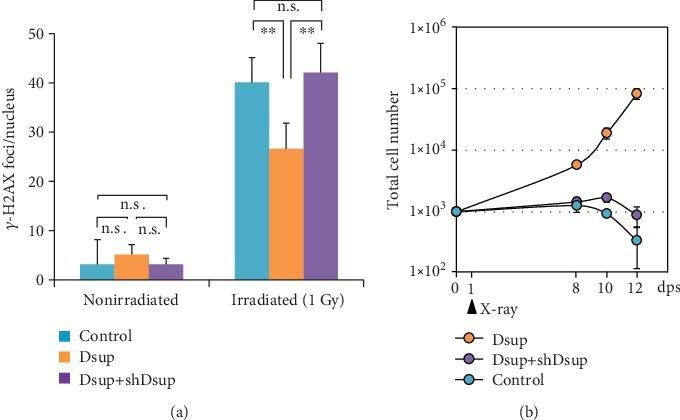
Dsup reduced X-ray-induced DNA damage (a) and improved viability of irradiated human cultured cells (b). The number of DNA-break marker, *γ*-H2AX foci in nonirradiated or 1 Gy-irradiated conditions (a), and growth curves after 4 Gy-irradiation (b) are compared among nonengineered human cultured cells (HEK293, control), Dsup-expressing cells (Dsup), and Dsup-knockdown cells (Dsup+shDsup). Reproduced from Hashimoto and Kunieda [253] under a Creative Commons Attribution-NonCommercial-ShareAlike 4.0 International License.

Unlike other radioresistant animals, tardigrades exhibit high radiotolerance either in a hydrated state or in a dehydrated state, suggesting the presence of specific machinery that relieves the indirect effects of radiation in this animal group. *Ramazzottius varieornatus* is one of the most radiotolerant species in tardigrades [244]. A recent study identified a tardigrade-unique DNA-associating protein, termed damage suppressor (Dsup) as a DNA-protecting agent from a chromatin fraction of *R. varieornatus* [251]. Intriguingly, in a human cultured cell line engineered to express the Dsup protein, DNA damage caused by X-ray radiation (1–10 Gy) was reduced to nearly half of those in nonengineered cells (Figure 4). In addition, Dsup can also reduce DNA fragmentation in human cells treated with H_2_O_2_ significantly. Thus, Dsup is capable of protecting DNA from both X-ray irradiation and attack by ROS. The ability of Dsup to protect DNA from ROS could explain the high radiation resistance of tardigrades even in wet conditions in which radiation causes biological damage *via* generation of ROS, which is known as indirect effects. After irradiation with a near lethal dose (4 Gy) of X-ray, nonengineered human cultured cells lose their proliferative ability (Figure 4), but surprisingly, Dsup-expressing cells survive the irradiation and even retain proliferative ability that is comparable with those of nonirradiated cells (Figure 4) [251]. Considering these results, Dsup is able to not only confer DNA protection but also improve radiotolerance to human cultured cells. A recent *in vitro* study also confirmed that Dsup can protect chromatin DNA from hydroxyl radicals [252]. Currently, a Dsup homolog has only been found in another tardigrade species, *Hypsibius exemplaris*, which belongs to the same taxonomic family of *R. varieornatus* [252–254]. These findings indicate that tardigrades have evolved their own stress-resistant machinery in their lineages and such unique machinery can also function in human cells. Radiation-resistant organisms including tardigrades are a valuable resource of undiscovered resistance genes and machineries, which might be used to enhance radiation resistance in other animal species including human.

## 9. Conclusions

In this review, discussion started with the environment of space radiation followed by a variety of simulated space radiation environments. Then, various adverse events by space radiation were discussed. In that chapter, state-of-the-art visualization technology of adverse events was discussed. Next, combined biological effects were discussed, and we reported that a newly developed 3D clinostat with synchronized irradiation capability would enable us to examine combined effects of radiation and *μG*. Radiation exposure management and radiation protection were then discussed. Finally, radioprotective organisms were presented because these organisms may aid the development of novel technologies that alleviate biological damage caused by radiation.

Understanding these topics in greater detail should facilitate better prediction of the risks and provide risk-mitigating strategies for future exploration space missions. In addition, meticulous use of available astronaut data, in particular long-duration mission crew members, should be beneficial. Furthermore, the use of rodent models in a Gateway program around the Moon orbit, for example, should provide important information required for a future human Mars mission.

## Figures and Tables

**Table 1:** Summary of brain cellular response to HZE irradiation (doses of less than 2 Gy).

Cells	Response	Irradiation	Dose rate (Gy/min)	Time after IR	Ref.
Neuron	Cell death	^56^Fe: 1.5 Gy	0.88	1 m	[141]
		^56^Fe: 1.6 Gy	1	12 m	[142]
	Deficits to proliferation and differentiation	^28^Si: 0.2, 1 Gy	1	24 h, 3 m	[143]
		^56^Fe: 0.3, 1 Gy	0.01–1	48 h, 1 m	[144]
	Changes to dendritic, axonal, and synaptic properties	GCR (H+He+O): 0.5 Gy	0.0616	100+ d	[145]
		^16^O, ^48^Ti: 0.05, 0.3 Gy	0.05, 0.25	15 w	[146]
		^1^H: 1 Gy	0.55	3 m	[147]
		^16^O, ^28^Si, ^4^He: 0.3 Gy		6 w	[148]
		^56^Fe: 0.5 Gy		3 m	[149]
		^1^H: 0.5 Gy + ^16^O: 0.1 Gy	^1^H: 0.18–0.19 ^16^O: 0.18–0.33	3 m	[150]

Glia	Astrocyte activation	^56^Fe: 1.6 Gy	1	12 m	[142]
	Microglial activation	GCR (H+He+O): 0.5 Gy	0.0616	100+ d	[145]
		^16^O, ^48^Ti: 0.05, 0.3 Gy	0.05, 0.25	15 w, 27 w	[146]
		^4^He: 0.05, 0.3 Gy	0.05	12 m	[151]

h: hours; d: days; w: weeks; m: months.

**Table 2:** Biological effects of radiation and *μG* in space experiments.

Interactive effects	Species	Biological index	Flight time	Irradiation^a^	Ref.
No	Human blood	Chromosomal aberration	12 h	+ ^32^P, *β*-rays	[167]
	*E. coli*, *S. cerevisiae*	DSB and SSB repair	14 d	Pre-X-rays	[168]
	*S. cerevisiae*	DSB repair	10 d	Pre-X-rays	[169]
	*E. coli*, *S. cerevisiae*	SOS response	2–4 d	Pre-X-rays	[170]
	Human blood	Chromosomal aberration	8 d	Pre- and post-*γ*-rays	[171]

Yes, ↑	*D. melanogaster*	Larval mortality	45 h	+ ^85^Sr, *γ*-rays	[172]
	*C. morosus*	Abnormality	7 d	No	[173]
	*S. cerevisiae*	DSB repair	9 d	No	[174]
	*D. melanogaster*	Mutation	8 d	No	[175]
	*D. discoideum*	Spore formation	9 d	No	[176]
	Human blood	Chromosomal aberration	10–485 d	Pre- and post-X-rays	[177]

Yes, ↓	*N. crassa*	Cell killing, mutation	45 h	+ ^85^Sr, *γ*-rays	[172]
	*D. radiodurans*	Cell killing	14 d	Pre-*γ*-rays	[178]

*E. coli*: *Escherichia coli*; *S. cerevisiae*: *Saccharomyces cerevisiae*; *D. melanogaster*: *Drosophila melanogaster*; *C. morosus*: *Carausius morosus*; *D. discoideum*: *Dictyostelium discoideum*; *N. crassa*: *Neurospora crassa*; *D. radiodurans*: *Deinococcus radiodurans*; DSB: DNA double-strand breaks; SSB: DNA single-strand breaks; h: hours; d: days. ^a^+ means simultaneous irradiation with spaceflight.

**Table 3:** Biological effects of radiation and simulated *μG* in ground experiments.

Interactive effects	Cells	Biological index	Devices	Irradiation^a^	Ref.
Yes, ↑	Lymphoblastoid	Mutation, micronuclei	RWV	Pre-^60^Co, *γ*-rays	[183]
	Lymphocyte	Mutation	RWV	Pre-^60^Co, *γ*-rays	[184]
	Lymphocyte	*γ*-H2AX	RWV	Pre-^137^Cs, *γ*-rays	[185]
	Lymphoblast	Apoptosis, ROS	RWV	Post-C-ion	[186]
	Fibroblast	Gene induction	RPM	+ ^252^Cf, neutron	[189]
	Neuron	Apoptosis, gene induction	RPM	+ ^252^Cf, neutron	[190]
	Fibroblast	Chromosomal aberration	SSS	+ X-rays, + C-ion	[193]
	Fibroblast	Cell cycle-promoting genes	SSS	+ C-ion	[194]

Yes, ↓	Lymphocyte	Apoptosis	RWV	Pre-^137^Cs, *γ*-rays	[187]
	Lymphoblastoid	Apoptosis	RWV	Pre-^60^Co, *γ*-rays	[183]
	Lymphocyte	Micro-RNA	RWV	Pre-^137^Cs, *γ*-rays	[188]
	Fibroblast	Cell cycle-suppressing genes	SSS	+ C-ion	[194]

ROS: reactive oxygen species; RWV: rotating wall vessel bioreactor; RPM: random positioning machine; SSS: system of simultaneous irradiation in simulated-microgravity. ^a^+ means simultaneous irradiation with spaceflight.

**Table 4:** Classification of tissues based on their radiosensitivity.

Frequency of cell division	Tissue	Radiosensitivity
++	Lymphoid tissue, hematopoietic tissue (bone marrow), testicular epithelium, follicular epithelium, and intestinal epithelium	Extremely high
+	Oropharyngeal oral epithelium, skin epidermis, hair follicle epithelium, sebaceous gland epithelium, bladder epithelium, esophageal epithelium, lens epithelium, gastric gland epithelium, and ureteral epithelium	High
+/–	Connective tissue, small vessel tissue, and growing cartilage/bone tissue	Intermediate
–	Mature cartilage/bone tissue, mucous serous epithelium, sweat gland epithelium, nasopharyngeal epithelium, lung epithelium, renal epithelium, liver epithelium, pancreatic epithelium, pituitary epithelium, thyroid epithelium, and adrenal epithelium	Low
– –	Nerve tissue and muscle tissue	Extremely low
